# Low SARS-CoV-2 Cq values in healthcare workers with symptomatic COVID-19 infections, regardless of symptom severity, The Netherlands, January to August 2022

**DOI:** 10.2807/1560-7917.ES.2023.28.4.2300007

**Published:** 2023-01-26

**Authors:** Carsten van Rossum, Corianne Meijer, Ingrid JM van Weerdenburg, Edmée C Bowles, Chantal P Rovers, Jaap ten Oever, Kim Stol, Nannet DJ van der Geest, Matthew B McCall, Alma Tostmann

**Affiliations:** 1 Radboud University Medical Centre, Radboud Centre for Infectious Diseases, Department of Medical Microbiology, Nijmegen, The Netherlands; 2 Radboud University Medical Centre, Radboud Centre for Infectious Diseases, Department of Internal Medicine, Nijmegen, The Netherlands; 3 Radboud University Medical Centre, Amalia Children’s Hospital, Department of Paediatrics, Division of Paediatric Infectious Diseases and Immunology, Nijmegen, The Netherlands; 4 Radboud University Medical Centre, Department of Occupational Health, Nijmegen, The Netherlands

**Keywords:** COVID-19, SARS-CoV-2, nosocomial infections, healthcare workers, PCR, Cq values, infectiousness

## Abstract

We analysed SARS-CoV-2 PCR Cq values from 3,183 healthcare workers who tested positive between January and August 2022. Median Cq values were lower in symptomatic than in asymptomatic HCW. The difference in Cq values between HCW with mild vs moderate/severe symptoms was statistically significant but negligibly small. To prevent nosocomial infections, all symptomatic HCW should be tested irrespective of symptom severity. This information can support decisions on testing and isolation, in the context of ongoing pressure on healthcare systems.

European national public health authorities still recommend healthcare workers (HCW) with symptoms indicative of coronavirus disease (COVID-19) to get tested and to refrain from work if positive. However, overall population immunity is high and the absolute individual risk of severe complications and hospitalisation – even in high-risk groups – has decreased compared with earlier phases of the pandemic due to vaccine-derived and/or natural immunity [1]. There has been increasing pressure to relax (existing) testing and isolation guidelines for HCW. However, this discussion should still be based on correct assumptions. Symptomatic infections are more infectious than asymptomatic infections [2]. However, in some clinical settings, it was discussed whether infections with mild symptoms would not require PCR testing assuming that mild infections would be less infectious that those with more severe symptoms. But this has not been investigated in studies.

We aimed to determine whether quantification cycle (Cq) values from severe acute respiratory syndrome coronavirus 2 (SARS-CoV-2) PCR, as a proxy for infectiousness [2], differed between HCW without, with mild or with moderate/severe early COVID-19 symptoms. 

## Assessment of SARS-CoV-2 PCR Cq values and early symptoms

We assessed Cq values and COVID-19 symptoms of HCW who tested positive for SARS-CoV-2 by qRT-PCR between 1 January and 30 August 2022 at a university hospital in the Netherlands [3]. During this period, according to the national guideline, HCW were obliged to do a PCR test if suffering from symptoms indicative of COVID-19 (even if they had a negative result in a self-applied rapid antigen test) or after high-risk exposure to a person with confirmed SARS-CoV-2 infection. HCW with a positive PCR test received a secured web-based questionnaire on the presence and onset of symptoms from the Occupational Health Department. We matched these answers to Cq values extracted from the medical microbiology laboratory information system. Data were analysed anonymously.

Omicron was the dominant SARS-CoV-2 variant in the Netherlands during the study period. We allocated the Omicron subvariants based on whole genome sequencing (WGS) results or, if WGS data were unavailable, on subvariant dominance in the Netherlands by date (BA.1: 1 January to 20 February 2022, BA.2: 21 February to 5 June 2022 and BA.4 and BA.5: 6 June to 30 August 2022) [4].

We defined the ‘severity’ of early COVID-19 symptoms by the likelihood of refraining from work due to these symptoms. Shortness of breath, fever and general malaise were categorised as ‘moderate/severe symptoms’, all other symptoms were categorised as ‘mild’ (incl. sore throat, sneezing, runny nose, headache). There were no ‘severe’ symptoms, as none reported symptoms that required immediate medical attention. 

## Early symptoms

In total, 3,183 HCW tested positive by SARS-CoV-2 PCR, of whom 2,822 (88.7%) reported COVID-19-related symptoms (Table). More than 95% reported one or more symptoms for which national COVID-19 testing guidelines recommend testing. 

**Table:** Characteristics and median Cq values in SARS-CoV-2 PCR of healthcare workers at a tertiary hospital in the Netherlands, January–August 2022 (n = 3,183)

Total	Number	Percentage	Median Cq value (25–75 percentile)	p value
	3,183	100	22.6 (20.1–26.7)	
**Age in years**				
19–30	1,161	36.5	23.1 (20.6–27.1)	p < 0.001
31–45	947	29.8	22.9 (20.4–27.2)	
46–60	836	26.3	21.9 (19.3–25.9)	
≥ 61	208	6.5	20.9 (19.0–23.6)	
Missing information	31	1.0	NA	
**Sex**				
Male	810	25.4	22.7 (20.0–26.5)	p = 0.765
Female	2,368	74.4	22.5 (20.1–26.8)	
Missing information	5	0.2	NA	
**Symptoms**				
Yes	2,822	88.7	22.2 (19.9–25.8)	p < 0.001
No	168	5.3	28.5 (23.8–34.1)	
Missing information	193	6.1	NA	
**Symptom severity (among symptomatic)**				
Mild	1,778	63.0	22.8 (20.2–26.6)	p < 0.001
Moderate/severe	1,044	37.0	21.4 (19.3–24.4)	
**Common symptoms^a^ (among symptomatic)**				
Common symptoms	2,720	96.4	22.2 (19.9–25.7)	p = 0.025
Uncommon symptoms	102	3.6	23.2 (20.6–28.9)	
**Omicron subvariant**				
BA.1	1,094	34.4	22.9 (20.1–27.5)	p < 0.001
BA.2	1,568	49.3	22.6 (20.2–26.5)	
BA.4/5	521	16.4	21.8 (19.6–25.7)	

COVID-19: coronavirus disease; NA: not applicable; SARS-CoV-2: severe acute respiratory syndrome coronavirus 2.

^a^ Symptoms were categorised as ‘common’ (cough, fever, runny nose, sore throat, ageusia/anosmia) and ‘uncommon’ (all other symptoms) based on the current community COVID-19 testing guidelines, where ‘common’ symptoms are the well-known indication for SARS-CoV-2 testing.

P values reflect the outcome of a Wilcoxon signed-rank test or a Kruskal–Wallis test, depending on the number of groups.

## SARS-CoV-2 PCR Cq values 

**Figure 1:**
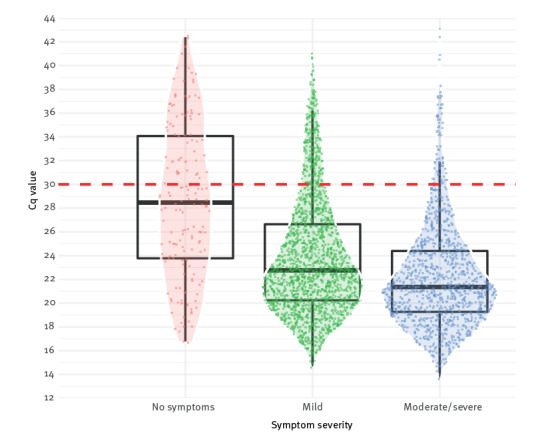
Distribution of Cq values of SARS-CoV-2 PCR in healthcare workers without and with mild or moderate/severe symptoms, January–August 2022 (n = 2,990)

**Figure 2:**
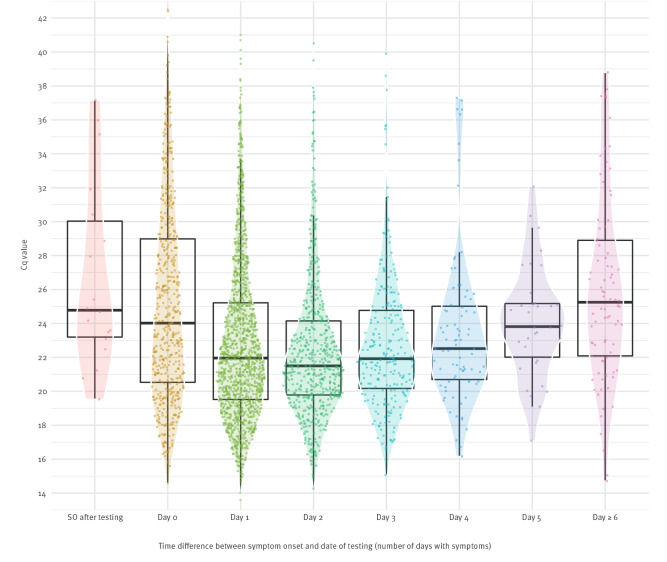
Cq values of symptomatic healthcare workers, by number of days between symptom onset and day of SARS-CoV-2 PCR testing, January–August 2022 (n = 2,872)

In 2,370 (84%) of symptomatic HCW, the symptoms had started between 2 days before testing and the day of testing. HCW with symptoms had lower median Cq values (22.2; interquartile range (IQR): 19.9–25.8) than HCW without symptoms (28.5; IQR: 23.8–34.1; p < 0.001; Mann–Whitney U test). There was a statistically significant yet negligible difference between HCW who reported mild symptoms (22.8; IQR: 20.2–26.6) and moderate/severe symptoms (21.4; IQR: 19.3–24.4; p < 0.001). The Cq values varied widely in all groups (Figure 1). The median Cq value was lowest in HCW who tested 1 or 2 days after symptom onset (Figure 2). When restricting the analysis to HCW whose symptoms started the day before or the day of testing, the difference in median Cq values between those with mild (n = 1,172) vs moderate/severe (n = 637) symptoms remained small (22.9; IQR: 20.2–27.4 or 21.3; IQR: 18.9–24.5, respectively).

The dashed line represents a Cq value of 30, above which infections were considered not infectious to be negligible.

Cq: quantification cycle; SARS-CoV-2: severe acute respiratory syndrome coronavirus 2; SO: symptom onset.

Median Cq values were 24.8, 24.0, 22.0, 21.5, 21.9, 22.5, 23.8 and 25.3 for healthcare workers who were tested 0, 1, 2, 3, 4, 5 or ≥ 6 days after symptom onset.

## SARS-CoV-2 variants

In total, 1,094 (34.4%) test results were allocated to Omicron BA.1, 1,568 (49.3%) to BA.2 and 521 (16.4%) to BA.4–5 (Table), and 742 (23.3%) of these allocations were based on WGS results. The difference in Cq values between symptomatic and asymptomatic HCW was consistent in the BA.1 (22.3 vs 29.1; p < 0.001) and BA.2 (22.4 vs 26.4; p < 0.001) group. There were only a few asymptomatic HCW in the BA.4–5 group due to changes in testing behaviour in that period. The difference in Cq value between HCW with mild and moderate/severe symptoms across the Omicron subvariant groups were consistent with the overall findings (data not shown).

## Discussion

We observed a large variation in the Cq values of SARS-CoV-2 PCR results in our study. The median Cq values were lower in HCW who reported symptoms compared with asymptomatically infected HCW. The Cq value difference between HCW with mild vs moderate/severe symptoms was small and, in our opinion, clinically negligible due to the large overlap in Cq values between these groups. We believe that if the objective of testing for SARS-CoV-2 in healthcare settings is to prevent nosocomial infections among patients, testing guidelines should include all symptomatic HCW for testing, regardless of symptom severity.

We used the SARS-CoV-2 PCR Cq values as a proxy for infectiousness [2]. Cq values above 30 are generally assumed to yield little culturable virus [5,6]. The majority of HCW in our study were considered infectious at the time of testing, and symptom severity alone could not be a reliable indicator of infectiousness, which is in line with observations in the literature [7,8].

The viral load decreased in HCW who were tested 3 days or more after reported symptom onset. This is helpful information in discussions on the work re-admission policies. The duration of infectiousness is shorter (varying from 5 to 10 days) when based on the presence of culturable virus than based on the presence of virus detectable by qRT-PCR [9,10]. While qRT-PCR positivity has been reported for a median of 28 days after symptom onset [11], studies show that no infectious virus was found 3–5 days after symptom resolution [12].

From April 2022 onwards, national testing policy changed in the Netherlands, and rapid antigen tests were included in the guidelines. Although our hospital testing guidelines did not officially change, HCW were less likely to perform a PCR following a negative rapid antigen test, as reflected in a lower frequency of PCR tests performed after April as the number of HCW tested decreased and the percentage of positive tests increased during this time (data not shown). This might explain the small number of asymptomatic infections we observed in the period of circulation of the Omicron BA.4/5 variant among our HCW. Another limitation is that symptoms were assessed only on the day of PCR testing and the onset of any symptoms thereafter remains unknown; some HCW may have been pre-symptomatic. Nor did we assess the duration of symptoms or potential worsening of symptoms after completing the questionnaire.

## Conclusion

Our findings contribute to the ongoing discussion on testing and isolation guidelines in healthcare settings and justify the current approach of COVID-19 testing upon symptoms, regardless of the severity of symptoms. The current influenza epidemic has led to additional pressure on healthcare and staff absences, and rational infection prevention policies regarding viral respiratory infections for HCW are still justified. With increased immunity in the population and reduced, but still existing, individual risks for COVID-19 complications in several patient groups, it is important to keep the right balance between preventing nosocomial infections and maintaining sufficient healthcare staff available for patient care. Infection prevention policies, such as universal or respiratory symptom-based masking in healthcare settings further reduce the risk of nosocomial respiratory infections regardless of the pathogen involved.
